# Huoxin pill prevents acute myocardial ischaemia injury via inhibition of Wnt/β‑catenin signaling

**DOI:** 10.1111/jcmm.17028

**Published:** 2021-11-16

**Authors:** Qing Wang, En Ma, Da Wo, Jinxiao Chen, Jia He, Jun Peng, Weidong Zhu, Dan‐ni Ren

**Affiliations:** ^1^ Fujian Key Laboratory of Integrative Medicine on Geriatric Academy of Integrative Medicine Fujian University of Traditional Chinese Medicine Fujian China; ^2^ Clinical and Translational Research Center Key Laboratory of Arrhythmias of Ministry of Education Research Institute of Heart Failure Shanghai East Hospital Tongji University School of Medicine Shanghai China

**Keywords:** Huoxin pill, ischaemia, Myocardial infarction, oxidative stress, Wnt/β‐catenin signalling

## Abstract

Myocardial infarction (MI) is one of the leading causes of death worldwide, and due to the widespread and irreversible damage caused, new therapeutic treatments are urgently needed in order to limit the degree of ischaemic damage following MI. Aberrant activation of Wnt/β‐catenin signalling pathway often occurs during cardiovascular diseases including MI, which results in excess production of reactive oxygen species (ROS) and further promotes myocardial dysfunction. Huoxin pill (HXP) is a Traditional Chinese Medicine formula that has been widely used in the treatment of coronary heart disease and angina; however, its mechanisms remain unclear. Here, we performed mouse models of MI and examined the effects and mechanisms of HXP in protecting against MI‐induced ischaemic damage. Our study showed that administration with HXP robustly protected against MI‐induced cardiac injuries, decreased infarct size and improved cardiac function. Moreover, HXP attenuated ischaemia‐induced DNA damage occurrence in vivo and H_2_O_2_‐induced DNA damage occurrence *in vitro*, via potent inhibition of adverse Wnt/β‑catenin signalling activation. Our study thus elucidated the role and mechanism of HXP in protecting against MI and oxidative stress‐induced injuries and suggests new therapeutic strategies in ischaemic heart disease via inhibition of Wnt/β‐catenin signalling pathway.

## 1 INTRODUCTION

Myocardial infarction (MI) remains one of the most common causes of deaths worldwide, accounting for 9 million deaths each year.^1^ At the onset of MI, occlusion of the coronary artery results in severely restricted blood flow to the myocardium, thereby resulting in widespread cardiomyocyte death and heart failure.^2^, ^3^ Current treatments of MI are mainly via physically promoting myocardial reperfusion in order to re‐establish coronary blood flow; however, severe and irreversible myocardial damage has often already occurred.^3^, ^4^ Thus, new therapeutic treatments are urgently required in order to limit the degree of ischemic damage following MI, as well as prevent pathological cardiac remodelling and subsequent progression to heart failure.

Wnt/β‐catenin signalling pathway plays a central role in embryonic development and tissue homeostasis^5^, ^6^, ^7^, ^8^; however, accumulating evidence has demonstrated its critical involvement in numerous diseases.^9^, ^10^, ^11^, ^12^, ^13^, ^14^ In the adult heart, the Wnt/β‐catenin signalling pathway is mostly turned off under normal conditions; however, it is adversely activated during cardiovascular diseases including coronary artery disease, myocardial ischaemia and heart failure.^13^, ^15^ Recent studies have demonstrated that Wnt/β‐catenin signalling pathway activation was deleterious for cardiac injury, while inhibition of Wnt/β‐catenin signalling was beneficial in ischaemic heart disease.^13^, ^15^, ^16^, ^17^, ^18^ Therefore, drugs that worked by inhibiting Wnt/β‐catenin signalling may be a viable strategy in protecting the heart from ischaemic injury.

Interestingly, adverse activation of Wnt/β‐catenin signalling following ischaemia is strongly linked with the enhanced production of reactive oxygen species (ROS) that results in DNA damage occurrence and serves as an important mediator of various pathophysiological signals involved in myocardial cell death.^19^, ^20^, ^21^ Excessive production of ROS promotes myocardial dysfunction and is evident in patients with acute MI and chronic heart failure.^22^ Indeed, oxidative stress‐induced DNA damage occurrence has been proposed as a key mechanism of cardiomyocyte death during cardiac ischaemia.^23^ Therefore, the inhibition of ROS via antioxidant drugs or agents that protected against oxidative stress is critical in preventing cardiac damage following MI.

Huoxin Pill (HXP) is a Traditional Chinese medicine (TCM) formula that is composed of the following ingredients: Panax ginseng, Ganoderma lucidum, Moschus, Aconiti Lateralis Radix Praeparata, Carthami Flos, Bovis Calculus Sativus, Margarita, bear bile, Bufonis Venenum and Borneolum Syntheticum and has been widely used as an alternative and complementary medicine for the treatment of coronary heart disease and angina pectoris throughout Asia.^24^ Numerous components in HXP, Ginsenosides from *Panax ginseng*, Ganoderic acids from *Ganoderma lucidum*, and flavonoids including Hydroxysafflor Yellow A from *Carthamus tinctorius L*. have been shown to exhibit antioxidative effects that may protect against cardiac injury^25^, ^26^, ^27^; however, the underlying mechanisms of HXP in the treatment of myocardial infarction remains largely unknown. Our current study aimed to examine the cardioprotective effects of HXP in preventing ischaemic damage and improving cardiac function following myocardial infarction, as well as elucidating the function of HXP in ameliorating oxidative stress‐induced DNA damage occurrence via inhibition of Wnt/β‐catenin signalling pathway.

## 2 MATERIALS AND METHODS

### 2.1 Materials

HXP (Batch: 15050101) was provided by Guangzhou Youcare Biopharmaceutics Co., Ltd. (Guangzhou, China). ANP (ab225844), BNP (ab236101), gamma H2A.X (γ‐H2A.X; ab81299), Cardiac Troponin T (ab8295), Anti‐DNA/RNA Damage antibody (ab62623), Goat polyclonal Secondary Antibody to Rabbit IgG ‐ H&L (Alexa Fluor^®^ 488), pre‐adsorbed (ab150081), Goat polyclonal Secondary Antibody to Mouse IgG ‐ H&L (Alexa Fluor^®^ 647), pre‐adsorbed (ab150119) was obtained from Abcam. Collagen‐I (14695–1‐AP), Collagen‐III (22734–1‐AP), ACTA‐1 (17521–1‐AP), ACTN2 (14221–1‐AP), GAPDH (60004–1‐Ig), TBP (22006–1‐AP) were purchased from Proteintech. β‐catenin (8480S), Phospho‐β‐Catenin (p‐β‐catenin; 9561S) were provided by CST. Goat anti‐Rabbit IgG Secondary Antibody (8715) and Goat anti‐Mouse IgG Secondary Antibody (6229) were purchased from SAB.

### 2.2 Preparation of HXP extract

To prepare the extract, HXP was grinded or pounded into a fine powder that passes through a sieve with nominal mesh aperture of 180 µm. The dried powder was re‐dissolved in PBS to a concentration of 10 mg ml^−1^, filtered with a 0.22 µm filter and stored at −20 °C for further use. For in vivo studies, HXP were used at final concentrations of 3 mg kg^−1^ (low‐dose group) or 9 mg kg^−1^ (high‐dose group). For in vitro studies, HXP was used at the indicated final concentrations.

### 2.3 Animals

Male C57BL/6 mice (8–12 weeks of age) were purchased from Shanghai SLAC Laboratory Animal Co., Ltd. (Shanghai, China). All animal studies were performed in accordance with institutional guidelines for the ethical care and use of laboratory animals and were approved by the University Committee on the Care and Use of Laboratory Animals at Fujian University of Traditional Chinese Medicine. Mice were randomly divided into 4 groups: (1) sham group; (2) MI + PBS group; (3) MI + HXP‐L (low‐dose group, 3 mg kg^−1^); (4) MI + HXP‐H (high‐dose group, 9 mg kg^−1^). Mice were anaesthetized by intraperitoneal injection of sodium pentobarbital (50 mg kg^−1^). HXP and PBS were administered via oral gavage every second day from one week prior to MI operation. Subsequently, MI was performed as previously described.^21^ Briefly, mouse was anaesthetized, and ligation of the proximal left anterior descending coronary artery was performed in all groups other than sham group that was subjected to the same procedure but without ligation. Mice were sacrificed via intraperitoneal injection of sodium pentobarbital (225 mg kg^−1^) euthanasia solution.

### 2.4 Two‐dimensional Echocardiography

Two‐dimensional echocardiography was performed using Visual Sonics Vevo 2100 Ultrasound machine (Toronto, ON). Mice were anaesthetized using 2% isoflurane supplemented with oxygen. M‐mode measurements were used to determine LV dimensions, including left ventricular internal dimension in diastole (LVID:d) and in systole (LVID:s). Left ventricular ejection fraction (EF) and fractional shortening (FS) were used to evaluate cardiac function. EF value is calculated by (LVIDd^3^ ‐ LVIDs^3^) / LVIDd^3^ × 100. FS value is calculated by (LVIDd ‐ LVIDs) / LVIDd^3^ × 100.

### 2.5 Quantitative Real‐time PCR

For quantitative real‐time PCR analysis, RNA was extracted using TRIzol reagent (Takara, Japan) and reverse transcribed to cDNA using a Prime Script RT reagent Kit according to the manufacturer's instructions (Takara, Japan). Real‐time quantitative PCR was performed with SYBR‐Green master mix in 96‐well optical plates using a Quant Studio 7 Flex Real‐Time PCR System (Applied Biosystems, USA). The primer sequences for ANP were 5'‐GGAGCCTACGAAGATCCAGC‐3’ (forward) and 5'‐TCCAATCCTGTCAATCCT ACCC‐3’ (reverse). The primer sequences for BNP were 5'‐CTTCGGTCTCAAGGCAGCAC‐3’ (forward) and 5'‐GCCCAAACGACTGACG GATC‐3’ (reverse). The primer sequences for ACTA‐1 were 5’‐ATGGATTCCCGTTCGAGTAC‐3’ (forward) and 5’‐TCAGCTGGATAGCGAC ATCG‐3’ (reverse). The primer sequences for Collagen‐I were 5'‐ATGGATTCCCGTTCGAGTAC‐3’ (forward) and 5'‐TCAGCTGGATAGCGAC ATCG‐3’ (reverse). The primer sequences for Collagen‐III were 5'‐CGTAGATGAATTGGGATGCA‐3’ (forward) and 5'‐ACATGGTTCTGGCTTC CAG‐3’ (reverse). The primer sequences for GAPDH were 5'‐TGGCCTTCCGTGTTCCTAC‐3’ (forward) and 5'‐GAGTTGCTGTTGAAGT CGCA‐3’ (reverse). GAPDH was used as the reference gene for determination of relative gene expressions. Relative fold changes were analysed using the comparative Ct (ΔΔCt) method and normalized to the control group.

### 2.6 Histological staining and Immunofluorescence

For histology analysis, hearts were fixed with 4% paraformaldehyde overnight, paraffin‐embedded, and sectioned (6 µm). Heart sections were stained with Masson's trichrome staining for visualization of infarcted area under a light microscope (Leica, Germany). Infarct size (%) was determined according to the ratio of endocardial infarct circumference compared with total left ventricular endocardial circumference ×100. For immunofluorescence, hearts were fixed with optimal cutting temperature (OCT) compound and frozen prior to sectioning. Slides were fixed in 4% paraformaldehyde, permeabilized with 0.25% Triton X‐100 in PBST (0.5% Tween in PBS), blocked with 5% bovine serum albumin (BSA) and incubated with the respective primary antibodies overnight at 4 °C. Following washing and incubation with secondary antibody, nuclei were stained with DAPI, and slides were mounted prior to imaging using a Zeiss LSM 710 confocal microscope.

### 2.7 TOPFlash/Renilla reporter gene assay and Cell Culture

TOPFlash/Renilla reporter gene assays were performed as previously described.^28^ 293T cells were co‐transfected with 1 ng ^−1^well V5‐tagged Wnt3a (Wnt3a), 40 mM LiCl, or 40 ng ^−1^well LRP6, as well as TOPFlash for 48 h prior to detection. RLU, relative light units. Negative control group was transfected with TOPFlash only. Adult ventricular cardiomyocytes AC16 cells were used for *in vitro* experiments. Cells were cultured in Dulbecco's modified Eagle's medium supplemented with 10% foetal bovine serum, 100 U ml^−1^ penicillin and 100 mg ml^−1^ streptomycin, and cultured in a 37 ˚C humidified incubator supplemented with 5% CO_2_. AC16 cardiomyocytes were pretreated with HXP (1 µg ml^−1^) or PBS for 2 hours, then stimulated with 100 µM of H_2_O_2_ for 30 minutes to model myocardial oxidative damage. For LiCl‐induced β‐catenin activation, cells were treated with 40 mM LiCl for 4 hours, then stimulated with 100 µM H_2_O_2_ for 30 minutes. For knockdown assays, cells were transfected with RNAiMAX and β‐catenin siRNA oligos or negative control siRNA as a control in OPTI‐MEM for 48 hours prior to collection.

### 2.8 Western blotting

Total protein was extracted with RIPA lysis buffer. Nuclear protein was extracted by nucleoprotein extraction kit (Sangon Biotech, China). Proteins were separated on 12% SDS‐PAGE gels and then transferred onto a 0.22 µm PVDF membrane, blocked with 5% non‐fat milk for 1 hour and incubated with the primary antibodies: anti‐β‐catenin, anti‐γ‐H2AX, anti‐TBP or GAPDH overnight at 4 ˚C. Subsequently, membranes were incubated with the secondary antibodies for 2 hours at room temperature and resulting protein bands were detected via chemiluminescence.

### 2.9 Statistical analysis

Statistical analysis was performed using SPSS 26.0 software (Chicago, IL, USA). Data were expressed as the mean ± standard error of mean (sem). Independent samples t test or Mann‐Whitney U test was performed on data with normal distribution or skewed distribution, respectively. P values <0.05 were considered statistically significant.

## 3 RESULTS

### 3.1 HXP improves cardiac function following ischaemic injury

**FIGURE 1:**
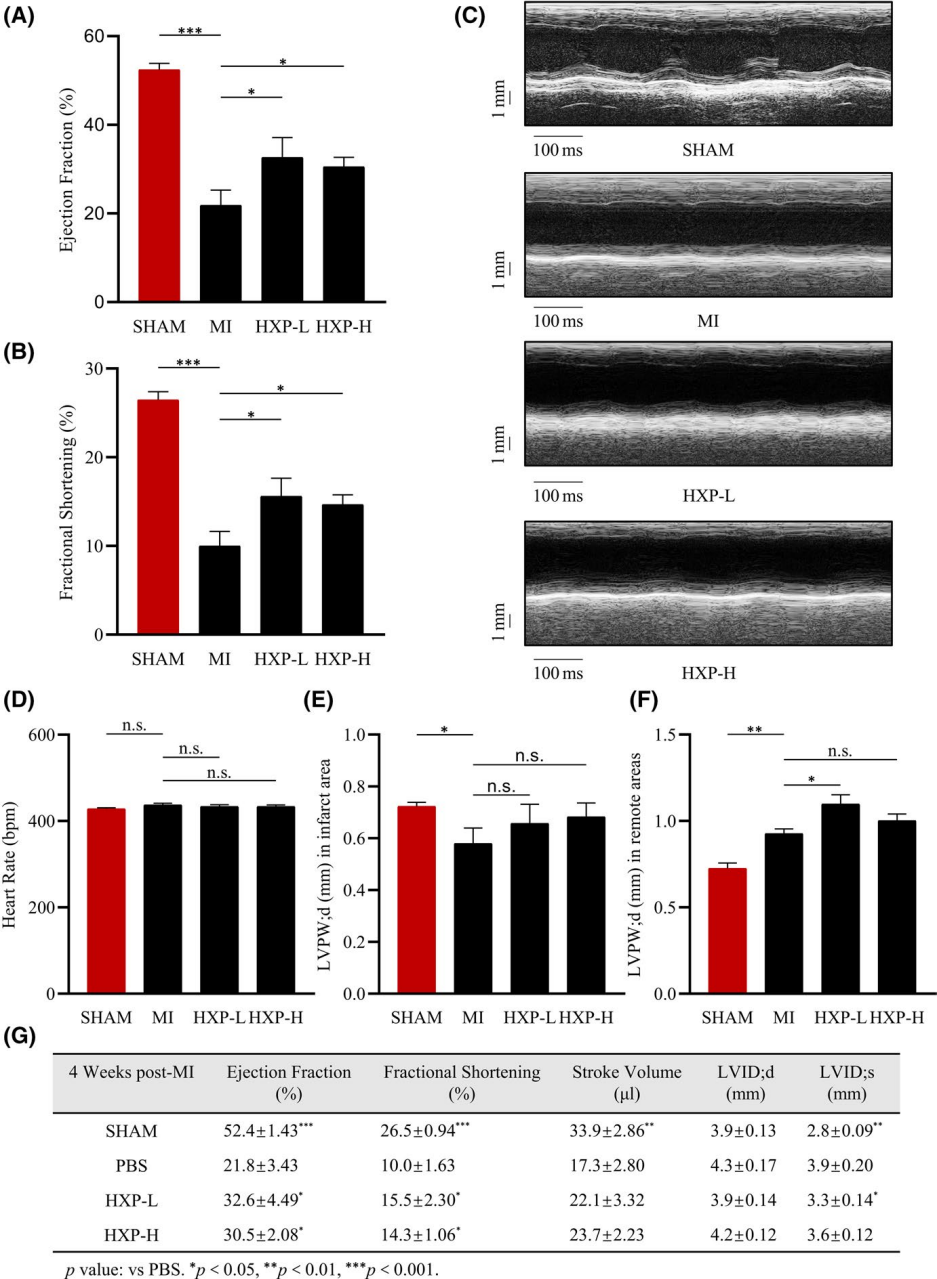
HXP improves cardiac function following ischaemic injury. (A‐B) Ejection fraction (EF%) and fractional shortening (FS%) parameters in mice administrated with PBS, low‐dose HXP‐L (3 mg kg d^−1^) or high‐dose HXP‐H (9 mg kg d^−1^) at 4 weeks post‐MI. *n* = 5 or more in each group, ^*^ *p* < 0.05, ^**^ *p* < 0.01, ^***^ *p* < 0.001. (C) Representative M‐mode echocardiographic images showing LV end‐systolic and end‐diastolic dimensions in mice administrated with PBS, low‐dose HXP‐L (3 mg kg d^−1^) or high‐dose HXP‐H (9 mg kg d^−1^) at 4 weeks post‐MI. (D‐G) Echocardiographic measurements of heart rate (D), LV end‐diastolic wall thickness in diastole at the infarct border zone (E), and remote zone (F), and cardiac function parameters (G) in sham, PBS, low‐dose HXP‐L (3 mg kg d^−1^), or high‐dose HXP‐H (9 mg kg d^−1^) mice at 4 weeks post‐MI. Data are presented as mean ± sem. ^*^ *p* < 0.05, ^**^ *p* < 0.01, ^***^ *p* < 0.001, n.s., no significance. *n* = 5 or more in each group. LVID;d—Left Ventricular Internal Dimension; diastolic; LVID;s—Left Ventricular Internal Dimension; systolic

In order to evaluate the cardioprotective effect of HXP following ischaemia, we performed mouse models of myocardial infarction (MI) in mice administered with either PBS or HXP at both low and high doses. Cardiac function was evaluated via echocardiography at 4 weeks following MI, which showed significant decreases in left ventricular (LV) ejection fraction (EF%), fractional shortening (FS%), as well as stroke volume (SV) values compared to SHAM‐operated mice (Figure 1A, B). Administration with HXP significantly improved cardiac function compared to PBS‐treated mice, with even a low dose of HXP having more significant cardioprotective benefits. Moreover, M‐mode echocardiography at the border of the infarct zone exhibited significant improvements in LV wall movement in HXP‐administrated mice compared to PBS‐administrated mice (Figure 1C) with equivalent heart rates between groups (Figure 1D). LV end‐diastolic wall thickness in diastole (LVED;d) was decreased at the zone bordering the infarct area following MI compared to SHAM‐operated mice (Figure 1E), which was to some extent attenuated in HXP‐treated mice. Interestingly, there was an increase in LVED;d wall thickness at the remote zone of LV near the papillary muscles at 4 weeks post‐MI, which was more pronounced in HXP‐treated mice, suggesting a protective over‐compensatory mechanism via increasing remote LV wall thickness post‐MI (Figure 1F). The summary of cardiac function parameters was shown in (Figure 1G). These results indicate that HXP has a robust effect in improving cardiac function following myocardial ischaemic injury.

### 3.2 HXP prevents MI‐induced cardiac remodelling

**FIGURE 2:**
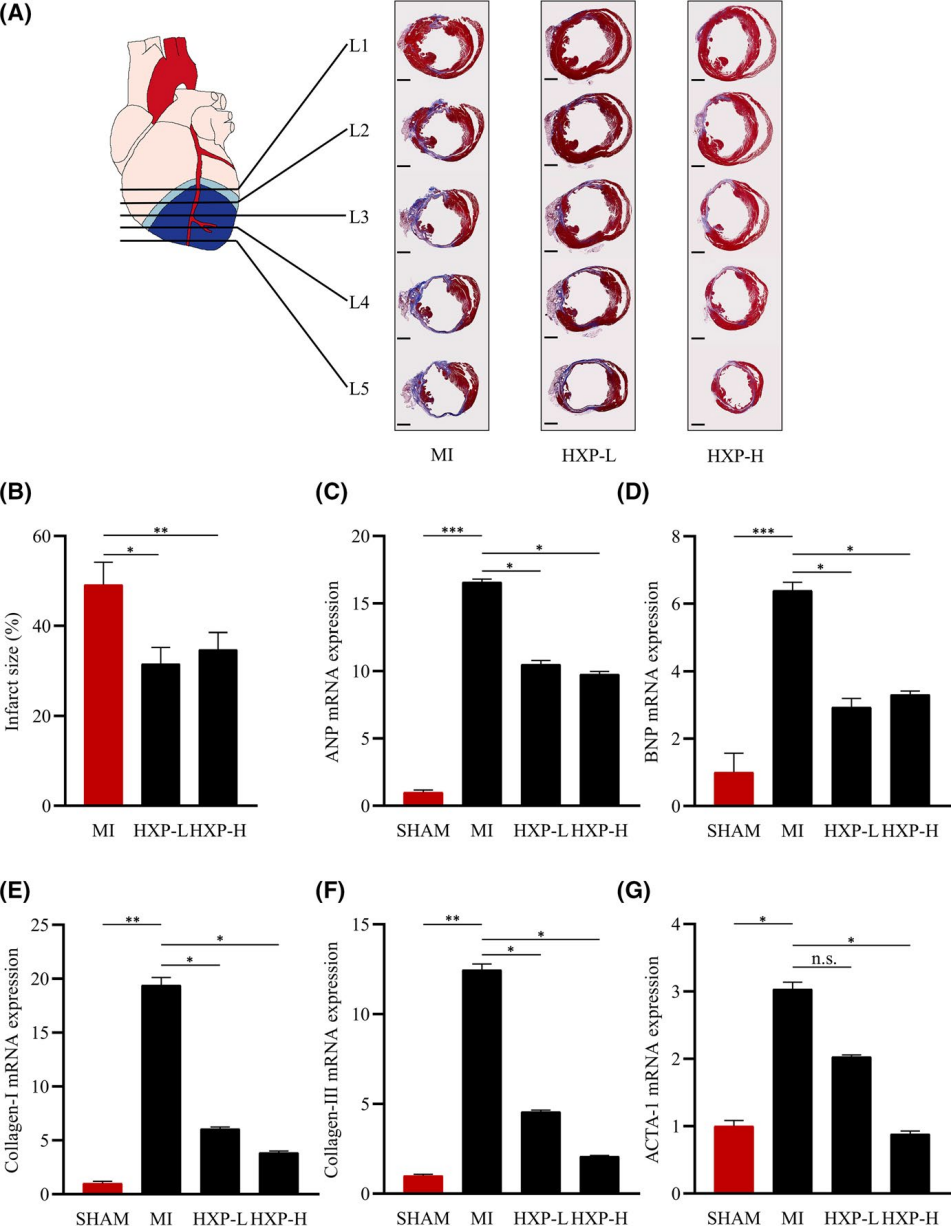
HXP prevents MI‐induced cardiac remodelling. (A) Masson's trichrome staining of hearts at 4 weeks post‐MI in mice administrated with PBS, low‐dose HXP‐L (3 mg kg d^−1^), or high‐dose HXP‐H (9 mg kg d^−1^) showing 5 different levels (L1‐L5) of heart transverse cross sections from the site of ligation towards the apex. Muscle fibres are stained red, and collagen‐rich fibrotic regions are stained blue. *n* = 9 or more for each group. Scale bar, 1 mm. (B) Quantification of infarct size in cardiac sections. Infarct size (%) = Mean endocardial infarct arc lengths at L3‐L4 divided by mean left ventricular endocardial arc lengths at L3‐L4 × 100. (C‐G) Relative mRNA expression levels of cardiac fibrosis genes (collagen‐I and collagen‐III) and heart failure genes (ANP, BNP, skeletal *α*‐actin) in the infarct regions of mice treatment with PBS, low‐dose HXP‐L (3 mg kg d^−1^) or high‐dose HXP‐H (9 mg kg d^−1^) at 2 weeks post‐MI. Data are presented as mean ± sem, n.s., no significance, ^*^ *p* < 0.05, ^**^ *p* < 0.01, ^***^ *p* < 0.01. *n* = 5 or more for each group

We further examined the degree of cardioprotection following myocardial infarction by examining the infarct size at 4 weeks following MI. Masson's trichrome staining showed that mice administrated with HXP (both low‐dose and high‐dose) had significant reductions in infarct size in the ischaemic heart compared to PBS‐administrated mice (Figure 2A, B). Notably, the infarct zones were generally reduced across all sectioning levels in HXP‐administered mice compared to PBS‐administered mice, indicative of a decreased degree of cardiac fibrosis across the whole heart following myocardial infarction.

Real‐time quantitative PCR analysis showed that the mRNA expressions of fibrosis markers Collagen‐I (Col‐I) and Collagen‐III (Col‐III), as well as heart failure markers atrial natriuretic peptide (ANP), brain natriuretic peptide (BNP) and skeletal α‐actin (ACTA‐1), were all significantly upregulated following MI (Figure 2C‐G). Administration with HXP resulted in significant reductions in the mRNA expressions of Col‐I, Col‐III, ANP, BNP and ACTA‐1 compared to PBS‐administered mice (Figure 2C‐G). Similarly, Western blot analysis showed that the protein expressions of these markers followed a similar trend of upregulation following MI, but were attenuated by HXP treatment (Supplemental Figure. 1A‐F). Interestingly, a low dose of HXP already provided adequate cardioprotective benefits, while a higher dose further improved several parameters, demonstrating the robust effect of HXP in protecting the heart against ischaemic injury.

### 3.3 HXP protects against ischaemia‐induced DNA damage in vivo and *in* vitro

**FIGURE 3:**
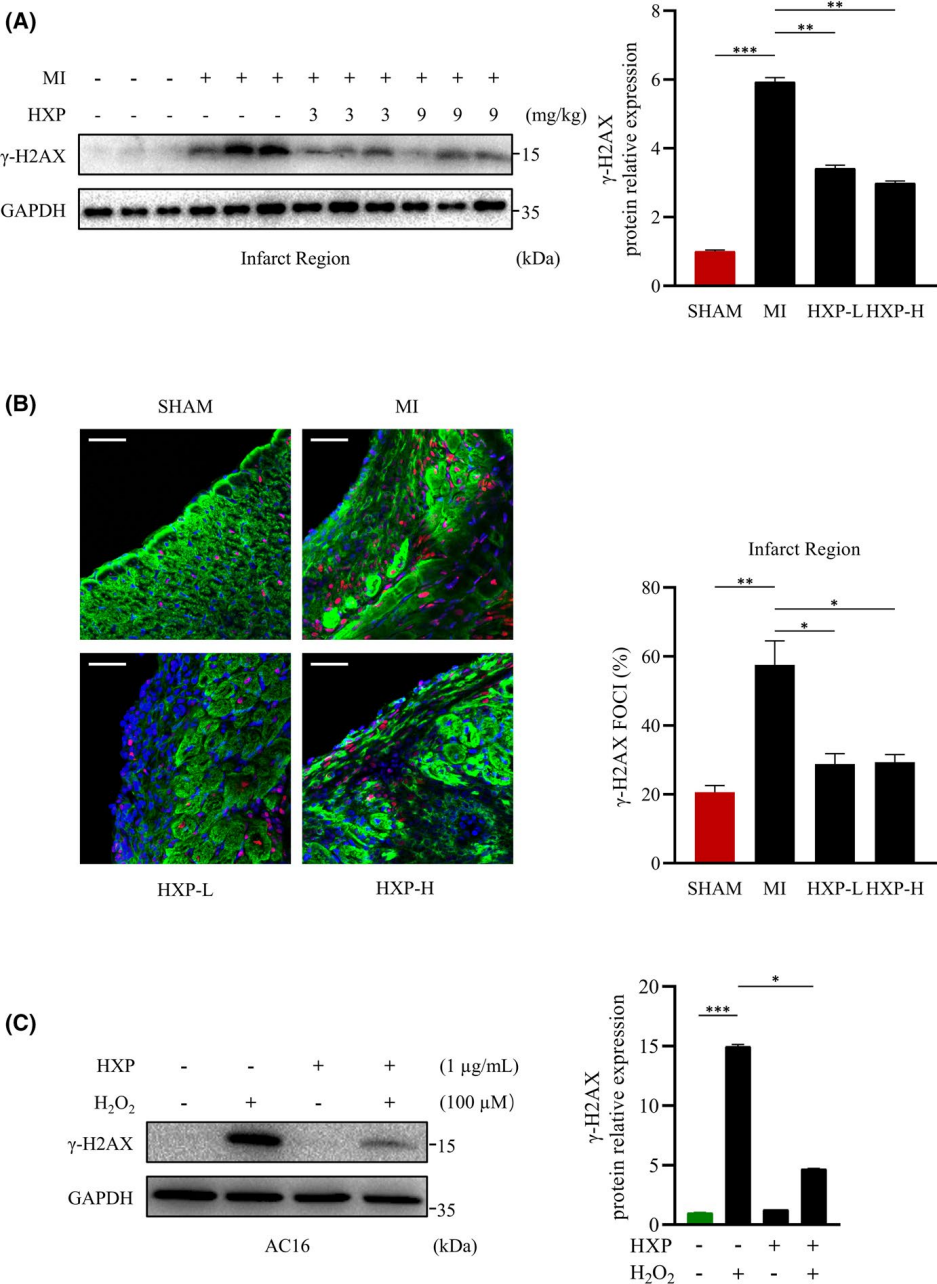
HXP attenuates ischaemia‐induced DNA damage in vitro and *in vivo*. (A) Representative immunoblots (left) and quantification (right) showing γ‐H2AX expression in ischaemic hearts from mice treated with PBS, low‐dose HXP‐L (3 mg kg d^−1^) or high‐dose HXP‐H (9 mg kg d^−1^) at 1‐week post‐MI. GAPDH was used as the loading control. Data are presented as mean ± sem, ^**^ *p* < 0.01, ^***^ *p* < 0.001. n = 3 for each group. (B) Representative immunofluorescence staining (left) and quantification (right) of γ‐H2AX foci (red), cardiac muscle marker Troponin T (green) and DAPI (blue) in the infarcted LV myocardium in SHAM‐operated mice and those treated with PBS, low‐dose HXP‐L (3 mg kg d^−1^) or high‐dose HXP‐H (9 mg kg d^−1^) at 1‐week post‐MI. Data are presented as mean ± sem, ^*^ *p* < 0.05, ^**^ *p* < 0.01. *n* = 4 for each group. Scale bar, 50 μm. (C) Representative immunoblot (left) and quantification (right) showing expression of γ‐H2AX following pretreatment with 1 µg ml HXP for 2 hours and treatment with 100 µM H_2_O_2_ for 30 minutes in AC16 cardiomyocytes. GAPDH was used as the loading control. Data are presented as mean ± sem, ^*^ *p* < 0.05, ^***^ *p* < 0.001 versus control group. *n* = 3 for each group

Reactive oxygen species (ROS) is widely known to play an important role in the development of cardiovascular diseases, in particular, during acute myocardial infarction. Unrepaired DNA damage and oxidative stress induced by ischaemia is the leading cause for cardiac cell death and is a common mechanism for acute or chronic heart diseases.^29^ Thus, we investigated the role of HXP in MI‐induced DNA damage. At 1 week following MI, the expression of γ‐H2AX, an early and sensitive marker of DNA damage was obviously upregulated in the infarct region of hearts compared to SHAM‐operated mice (Figure 3A). Notably, mice administered with HXP had markedly reduced levels of γ‐H2AX compared to PBS‐administered mice in the infarct region post‐MI, as shown via WB (Figure 3A), and percentage of γ‐H2AX positive foci via immunofluorescence (Figure 3B), demonstrating the ability of HXP in protecting against ischaemic‐induced DNA damage following MI. Furthermore, the degree of nuclear ROS following MI was examined via immunofluorescence staining of the infarcted LV myocardium using 8‐Oxo‐7,8‐dihydro‐2'‐deoxyguanosine (oxo‐8‐dG) antibody for oxidative DNA damage (Supplemental Figure 2A). There was a significant increase in the numbers of oxo‐8‐dG foci surrounding the nucleus in the infarcted LV myocardium post‐MI, indicative of oxidative stress‐induced nuclear ROS generation, which was significantly decreased in HXP‐treated mice, demonstrating that HXP protected against oxidative stress‐induced nuclear ROS following myocardial infarction.

Hydrogen peroxide (H_2_O_2_) is a source of ROS widely used to mimic oxidative stress‐induced damage in vitro. Indeed, treatment with H_2_O_2_ significantly and rapidly activated (as early as 15 min and peaking at1 hour following H_2_O_2_ treatment) γ‐H2AX expression in human AC16 cardiomyocytes (Supplemental Figure 3A, B). We thus used H_2_O_2_ treatment to model the occurrence of cellular oxidative damage in vitro. Pretreatment with HXP did not alter the basal expressions of γ‐H2AX, but surprisingly, HXP pretreatment significantly attenuated H_2_O_2_‐induced expression of γ‐H2AX in AC16 cardiomyocytes (Figure 3C), suggesting that HXP prevents H_2_O_2_‐induced DNA damage occurrence in vitro. Taken together, these results suggest that HXP protects against ischaemic cell damage by attenuating ischaemia‐induced DNA damage.

### 3.4 HXP prevents ischaemia‐induced cardiac injury and DNA damage via potent inhibition of Wnt/β‑catenin signalling

**FIGURE 4:**
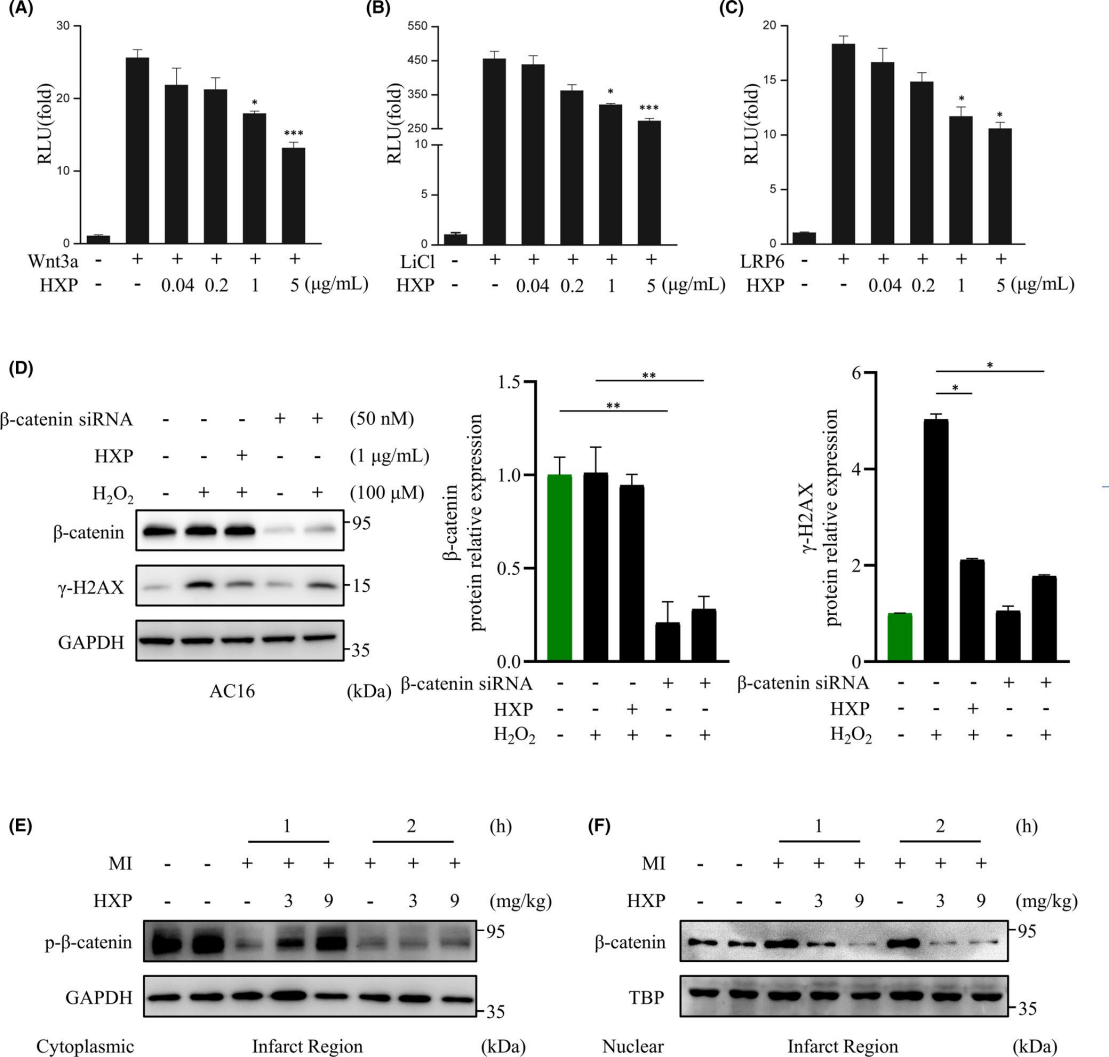
HXP is a potent inhibitor of Wnt/β‑catenin activation‐induced cardiac injury and DNA damage. (A‐C) Reporter gene assay using TOPflash analysis. Wnt3a (A), LiCl addition (B), or full‐length LRP6 (C) were transfected with TOPflash in HEK293 cells for 48 h. HXP or LiCl were directly added to the transfected cells at varying concentrations, as indicated. RLU, relative light units. Values are presented as the mean ± sem. n.s., no significance, ^*^ *p* < 0.05, ^***^ *p* < 0.001 versus control group. *n* = 3 for each experiment. (D) Representative immunoblot (left) and quantification (right) showing expressions of γ‐H2AX and β‐catenin following siRNA‐mediated knockdown of β‐catenin for 48 hours, subsequent pretreatment with 1 µg ml^−1^ HXP for 2 hours and treatment with 100 µM H_2_O_2_ for 30 minutes. GAPDH was used as the loading control. Data are presented as mean ± sem, ^*^ *p* < 0.05, ^**^ *p* < 0.01. n = 3 for each. (E, F) Representative immunoblot showing the expression of cytoplasmic phosphorylated‐β‐catenin (E) and nuclear β‐catenin (F) in the infarct region of mice treated with PBS, low‐dose HXP‐L (3 mg kg d^−1^), or high‐dose HXP‐H (9 mg kg d^−1^) at 1 h and 2 h post‐MI. GAPDH was used as the cytoplasmic loading control. TBP was used as the nuclear loading control

There is accumulating evidence that suggests Wnt/β‐catenin signalling pathway plays a central role in ischaemia‐induced cardiac injury. To further investigate the underlying mechanism by which HXP prevents ischaemic injury and oxidative stress‐induced DNA damage, we examined the effect of HXP on the activation of Wnt/β‐catenin pathway using β‐catenin‐responsive TOPflash gene assays. Wnt/β‑catenin signalling was significantly activated upon stimulation with Wnt ligand Wnt3a, Wnt agonist LiCl and Wnt receptor LRP6 (Figure 4A‐C). Surprisingly, HXP administration exhibited a potent and specific inhibitory effect on the activation of TOPFlash in a dose‐dependent manner, demonstrating that HXP is a robust agent in inhibiting the activation of Wnt/β‐catenin signalling pathway (Figure 4A‐C).

We next performed siRNA knockdown assays of β‐catenin, which showed that following H_2_O_2_ treatment, knockdown of β‐catenin significantly attenuated γ‐H2AX expression in AC16 cardiomyocytes, to a similar extent as HXP treatment (Figure 4D), demonstrating that Wnt/β‐catenin pathway plays a key role in H_2_O_2_‐induced damage. Similarly, we investigated the effect of HXP on the expression of β‐catenin following MI. It is well known that the levels of nuclear β‐catenin are rapidly upregulated following myocardial ischaemia; however, in the basal state, β‐catenin is constantly phosphorylated and targeted for degradation in the cytoplasm. Our results showed that the levels of cytoplasmic phosphorylated β‐catenin in the infarct region of hearts were markedly downregulated from as early as 1 h and 2 h following MI (Figure 4E), whereas the levels of nuclear β‐catenin were significantly upregulated at these timepoints (Figure 4F), demonstrating the accumulation and therefore activation of Wnt/β‐catenin signalling pathway post‐MI. Similar to the infarct region, the expression of nuclear beta‐catenin was significantly elevated following MI in the non‐infarct remote region, demonstrating that the accumulation and activation of Wnt/beta‐catenin signalling post‐MI are evident throughout the entire heart (Supplemental Figure 4A). Notably, HXP‐treated mice reduced the levels of nuclear beta‐catenin accumulation compared to PBS‐administered mice following MI even in the remote region, although this effect was slightly less pronounced than in the infarcted region. Notably, mice administered with HXP had markedly higher levels of cytoplasmic phospho‐β‐catenin, but reduced levels of nuclear β‐catenin accumulation compared to PBS‐administered mice in a dose‐dependent manner in both the infarct region (Figure 4E, F) and the remote region (Supplemental Figure 4A, B), demonstrating that HXP can significantly inhibit MI‐induced activation of Wnt/beta‐catenin signalling not only in the infarcted myocardium, but throughout the entire heart.

**FIGURE 5:**
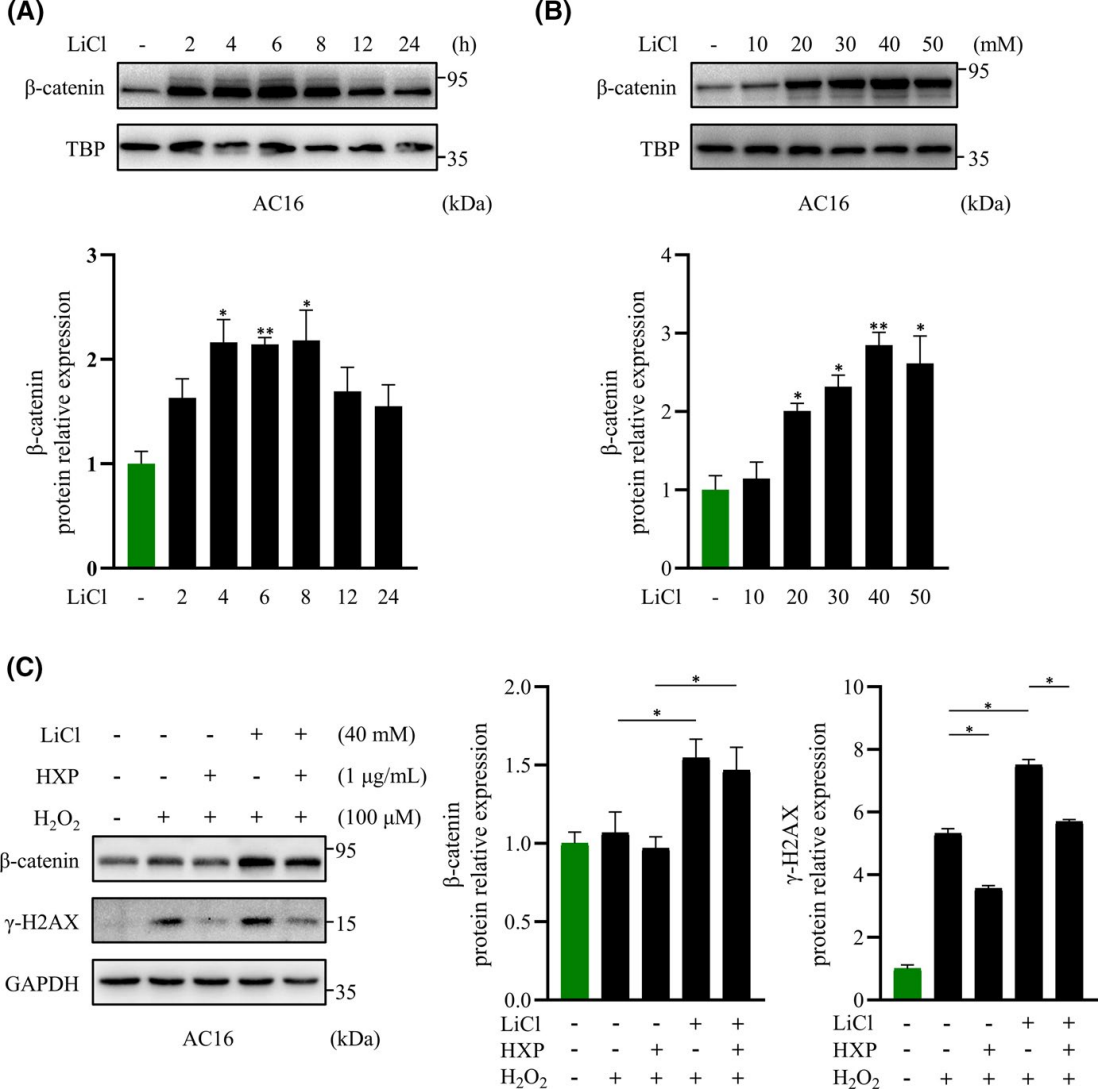
HXP attenuates H_2_O_2_‐induced DNA damage via inhibition of Wnt/β‐catenin pathway. (A) Representative immunoblot (top) and quantification (bottom) showing the expression of nuclear β‐catenin following LiCl stimulation at different time intervals in AC16 cardiomyocytes. TBP was used as the loading control. n.s., no significance, ^*^ *p* < 0.05, ^**^ *p* < 0.01 versus control group. *n* = 3 for each experiment. (B) Representative immunoblot (top) and quantification (bottom) showing the expression of nuclear β‐catenin following LiCl stimulation at different concentrations in AC16 cardiomyocytes. TBP was used as the loading control. n.s., no significance, ^*^ *p* < 0.05, ^**^ *p* < 0.01 versus control group. *n* = 3 for each experiment. (C) Representative immunoblot (left) and quantification (right) showing the expression of γ‐H2AX and β‐catenin following pretreatment with 1 µg ml^−1^ HXP for 2 hours, 40 mM LiCl for 4 hours, and subsequent treatment with 100 µM H_2_O_2_ for 30 minutes. GAPDH was used as the loading control. Data are presented as mean ± sem, ^*^ *p* < 0.05. n = 3 for each

To further investigate the role of HXP in oxidative stress‐induced damage via inhibition of Wnt/β‑catenin signalling, we used LiCl, a stabilizer of β‐catenin that acts by inhibiting the phosphorylation and degradation of cytoplasmic β‐catenin. Indeed, treatment with LiCl significantly and rapidly activated the level of nuclear β‐catenin in AC16 cardiomyocytes (Figure 5A), and in a dose‐dependent manner (Figure 5B). Notably, LiCl significantly augmented the expression of γ‐H2AX induced by H_2_O_2_ treatment in AC16 cardiomyocytes, which was attenuated by pretreatment with HXP (Figure 5C), suggesting that HXP can prevent H_2_O_2_‐induced DNA damage via inhibition of Wnt/β‐catenin pathway. Taken together, these results demonstrate that the effect of HXP in protecting against ischaemic and oxidative stress‐induced damage was via robust inhibition of Wnt/β‑catenin signalling pathway.

## 4 DISCUSSION

Acute myocardial infarction is the leading cause of death worldwide.^30^ There is accumulating evidence that suggests Wnt signalling pathway is activated during the wound healing process following MI. Studies have demonstrated that injections of recombinant Wnt3a following MI led to a worsened cardiac function and increased infarct size.^31^ Moreover, stabilization of cardiomyocyte β‐catenin expression further deteriorated cellular function following cardiac insults.^17^ In contrast, inhibition of Wnt pathway, including secreted Frizzled‐related proteins,^32^, ^33^, ^34^, ^35^ small molecular inhibitors such as SEN195, pyrvinium, WNT974 or ICG001 have been shown to alleviate pathological remodelling and improve cardiac function following MI.^18^, ^36^, ^37^, ^38^ Further, our previous study demonstrated that inhibition and cardiac‐specific deletion of β‐catenin protected the heart from ischemic injury.^17^ Inhibition of Wnt signalling pathway should have therefore a general protective effect following myocardial infarction, and drugs that can minimize cardiac ischaemic injury via inhibiting adverse activation of Wnt/β‐catenin signalling is urgently needed.

Numerous studies have demonstrated that increased reactive oxygen species (ROS) promotes myocardial dysfunction and cardiac damage, which can lead to heart failure.^22^ Increased ROS has also been shown to exacerbate cardiomyocyte damage and apoptosis following MI.^39^ Due to the fact that cardiac ischaemia triggers rapid release of ROS that subsequently leads to widespread DNA damage, the inhibition of ROS is crucial in attenuating cardiac damage following MI. Various antioxidant drugs have shown promising effects in protecting against MI‐induced cardiac damage both in animal models and clinically.^40^, ^41^ Activation of Wnt/β‐catenin signalling has also been shown to enhance ROS production, while its inhibition not only prevents ROS, but also had protective effects following MI.^17^ Thus, the ability of HXP to not only inhibit Wnt/β‐catenin signalling activation following MI, but also prevent ROS production, demonstrated its effectiveness in the treatment of cardiac ischaemia.

Due to the complex pathogenesis and limited effective treatment options for acute MI, there is still an urgent need for drugs that can minimize cardiac ischemic injury or improve cardiac function following MI. Traditional Chinese medicine (TCM) has been widely used for the treatment of various cardiovascular diseases in China and numerous Asian countries. One such TCM prescription is HXP, which has been clinically used in the treatment of coronary heart diseases.^24^ Our current study demonstrated for the first time that HXP acts as a robust inhibitor of Wnt/β‐catenin signalling, thereby strongly protecting against ischaemic damage and improving cardiac function following MI. Notably, HXP administration not only attenuated cardiac ischaemic damage and scar formation, it also resulted in drastic improvements in cardiac function. These findings provide important insights into the roles and mechanisms of HXP, and due to the ease of HXP drug application via oral intake implicate its use for the clinical treatment of myocardial infarction.

## CONFLICT OF INTEREST

The authors confirm that there are no conflicts of interest.

## AUTHOR CONTRIBUTIONS

**Qing Wang:** Formal analysis (equal); Investigation (lead); Visualization (equal); Writing‐original draft (lead). **En Ma:** Investigation (equal); Methodology (lead); Writing‐review & editing (equal). **Da Wo:** Data curation (lead); Formal analysis (equal); Investigation (equal); Visualization (equal); Writing‐review & editing (lead). **Jinxiao Chen:** Resources (equal); Software (equal). **Jia He:** Resources (equal); Software (equal). **Jun Peng:** Resources (lead). **Weidong Zhu:** Resources (lead); Supervision (equal). **Dan‐ni Ren:** Conceptualization (lead); Funding acquisition (lead); Supervision (lead).

## Supporting information

Fig S1‐4

## Data Availability

The data that support the findings of this study are available from the corresponding author upon reasonable request.
