# Supplementary figures and images for: Huoxin pill prevents acute myocardial ischaemia injury via inhibition of Wnt/β‑catenin signaling
Source: J Cell Mol Med. 2021 Nov 16;25(24):11053–62. doi: 10.1111/jcmm.17028 (PMC8650034; doi:10.1111/jcmm.17028)

Supplemental Fig. 1

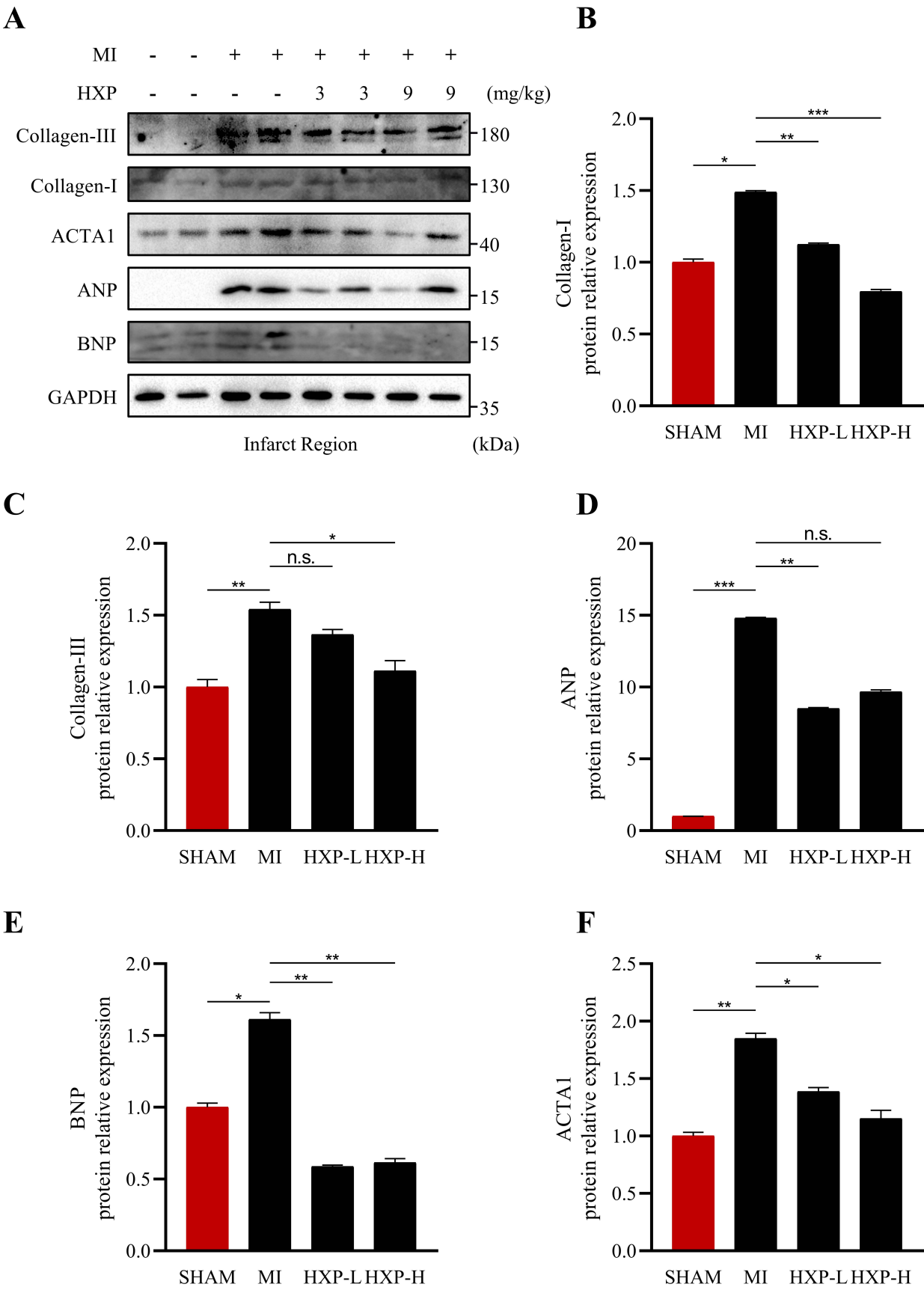

Supplemental Fig. 2

A

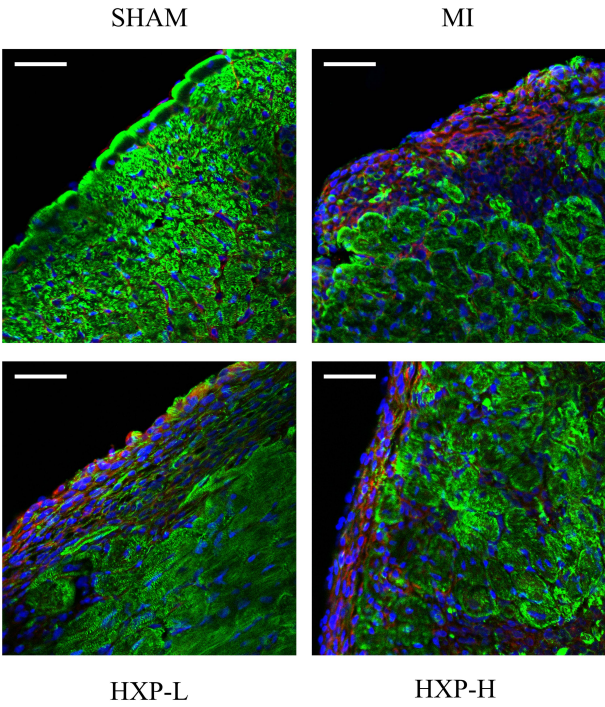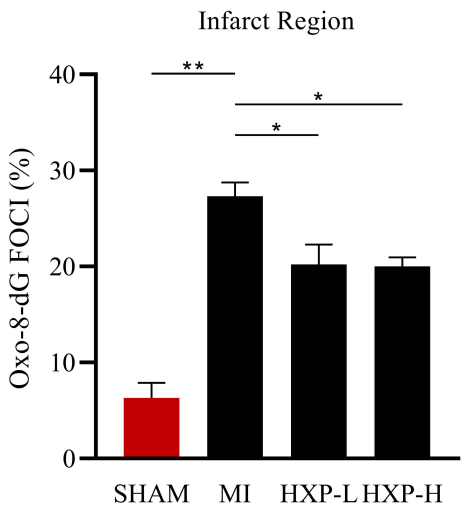

Supplemental Fig. 3

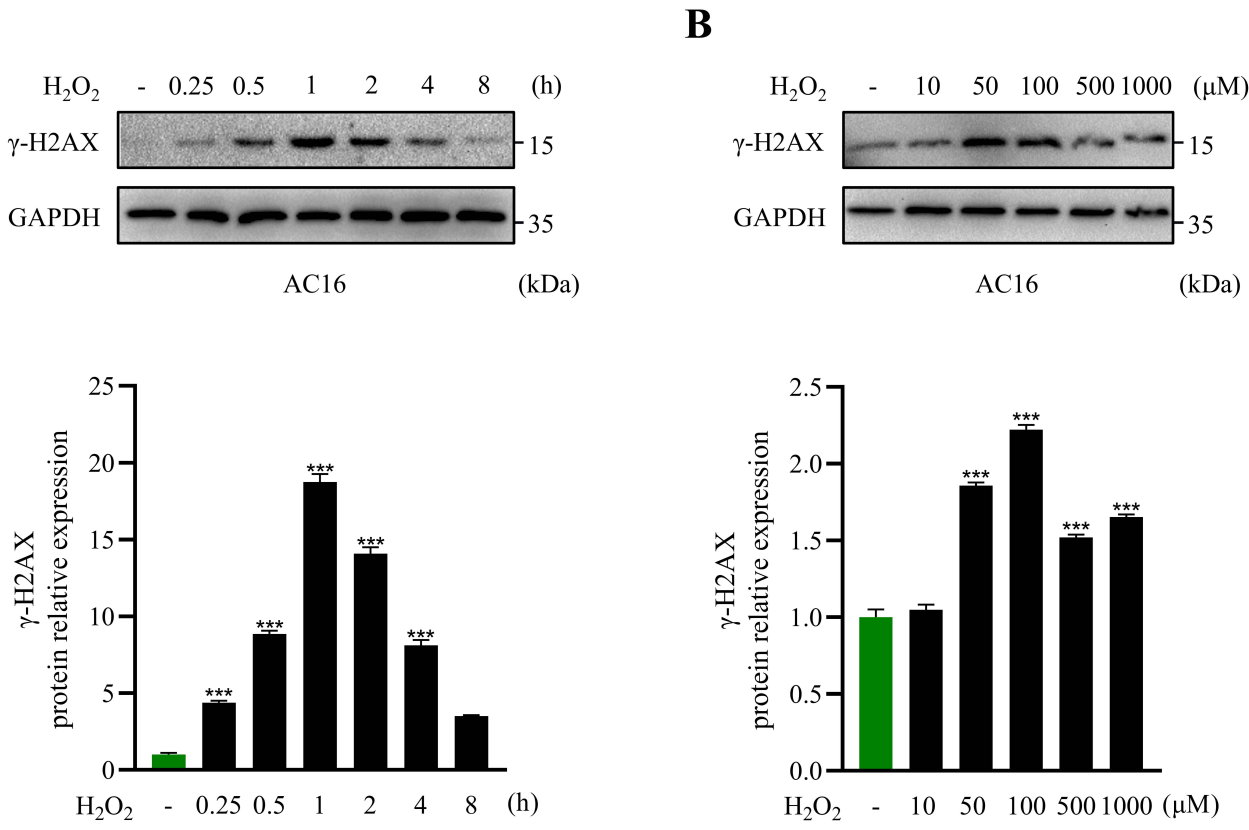

Supplemental Fig. 4

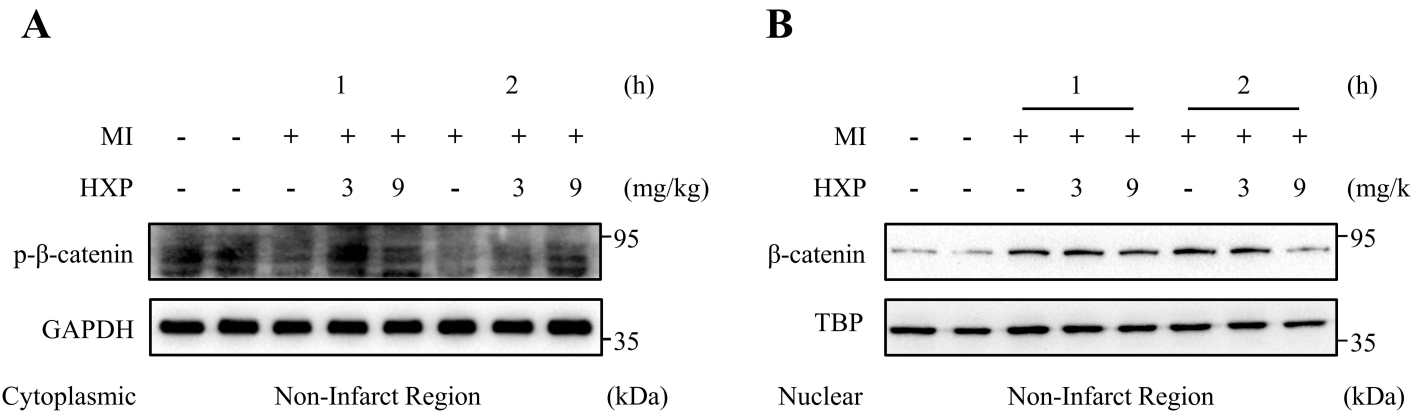

Supplement: Supplementary file 1 — Fig S1‐4 [file JCMM-25-11053-s001.pdf]
